# Comprehensive analysis of flavohemoprotein copy number variation in *Giardia intestinalis:* exploring links to metronidazole resistance

**DOI:** 10.1186/s13071-024-06392-5

**Published:** 2024-08-10

**Authors:** Vlasta Korenková, Filip Weisz, Aneta Perglerová, Simone M. Cacciò, Eva Nohýnková, Pavla Tůmová

**Affiliations:** 1 Institute of Immunology and Microbiology, 1st Faculty of Medicine, Charles University, Prague, Czech Republic; 2 Department of Infectious Diseases, Istituto Superiore Di Sanita, Rome, Italy

**Keywords:** *Giardia intestinalis*, Metronidazole, Copy number variation, Digital PCR, Aneuploidy, Chromosomes, Flavohemoprotein, Flavohemoglobin

## Abstract

**Background:**

Giardiasis, caused by the protozoan parasite *Giardia intestinalis*, often presents a treatment challenge, particularly in terms of resistance to metronidazole. Despite extensive research, markers for metronidazole resistance have not yet been identified.

**Methods:**

This study analysed 28 clinical samples of *G. intestinalis* from sub-assemblage AII, characterised by varying responses to metronidazole treatment. We focussed on copy number variation (CNV) of the multi-copy flavohemoprotein gene, analysed using digital polymerase chain reaction (dPCR) and next generation sequencing (NGS). Additionally, chromosomal ploidy was tested in 18 of these samples. Flavohemoprotein CNV was also assessed in 17 samples from other sub-assemblages.

**Results:**

Analyses revealed variable CNVs of the flavohemoprotein gene among the isolates, with no correlation to clinical metronidazole resistance. Discrepancies in CNVs detected from NGS data were attributed to biases linked to the whole genome amplification. However, dPCR helped to clarify these discrepancies by providing more consistent CNV data. Significant differences in flavohemoprotein CNVs were observed across different *G. intestinalis* sub-assemblages. Notably, *Giardia* exhibits a propensity for aneuploidy, contributing to genomic variability within and between sub-assemblages.

**Conclusions:**

The complexity of the clinical metronidazole resistance in *Giardia* is influenced by multiple genetic factors, including CNVs and aneuploidy. No significant differences in the CNV of the flavohemoprotein gene between isolates from metronidazole-resistant and metronidazole-sensitive cases of giardiasis were found, underscoring the need for further research to identify reliable genetic markers for resistance. We demonstrate that dPCR and NGS are robust methods for analysing CNVs and provide cross-validating results, highlighting their utility in the genetic analyses of this parasite.

**Graphical Abstract:**

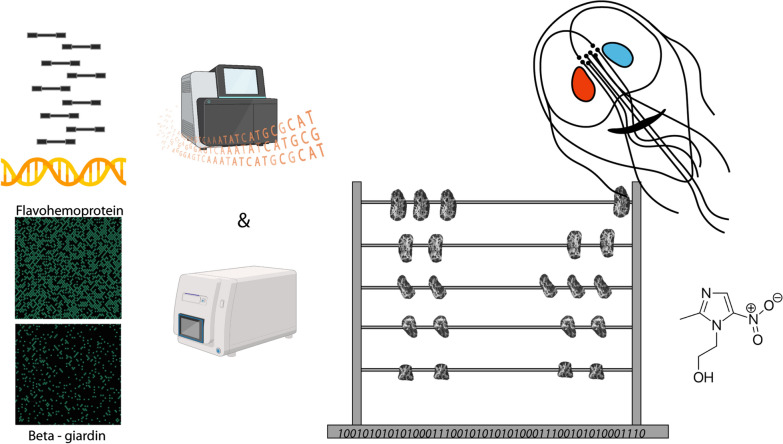

**Supplementary Information:**

The online version contains supplementary material available at 10.1186/s13071-024-06392-5.

## Background

*Giardia intestinalis* is a single-celled intestinal parasite with a compact genome ranging in size from 10.5 Mbp to 13.2 Mbp [1, 2]. It lacks typical organelles such as the Golgi apparatus, peroxisomes, and has highly reduced mitochondria without standard mitochondrial functions [3]. *Giardia* possesses two diploid nuclei of equal size and is therefore classified as a tetraploid organism. Despite the similarity in their genomic content, the two nuclei exhibit constitutive aneuploidy, leading to unequal genetic distribution [4, 5]. Its life cycle includes stages where the ploidy status varies: cysts are octoploid while trophozoites are tetraploid [6–8].

*G. intestinalis* has long interested researchers due to its striking genetic diversity. The classification of this intestinal parasite into eight assemblages (A-H) and subsequent subdivision into sub-assemblages provided a framework for understanding its genetic landscape [9–12]. This subdivision often relies on the genotyping of a few unlinked marker genes [13, 14]. The taxonomic status of these assemblages is debated as they could represent distinct species [15–17].

The genome of *Giardia* usually comprises five diploid chromosomes in each nucleus, with approximately 5000 protein-coding genes identified, accounting for about 92% of all genes, most of them being single-copy genes [18]. Noncoding and repetitive sequences represent only a small fraction of the genome, with a few introns identified thus far [19, 20]. *Giardia* also possesses multi-copy coding genes organized into multigene families distributed along its five chromosomes. The most significant among them is the variable surface protein (VSP) gene family, which makes up approximately 4% of the coding fraction of the genome. VSP genes are responsible for the expression of VSP cysteine-rich antigens on the surface of trophozoites, allowing the parasite to escape recognition through the host’s adaptive immune system [21].

Another important multi-copy gene is *hmpA*, which encodes a Flavohemoprotein enzyme, also known as Flavohemoglobin [22]. It belongs to the heme protein family containing electron transfer proteins [23]. This enzyme is the most significant nitric oxide (NO)-metabolizing protein, operating effectively as NO dioxygenase under microaerophilic conditions [24]. Flavohemoprotein is expressed and up-regulated in response to higher levels of host-derived NO, enabling the trophozoites to metabolize free radical NO into harmless nitrate using oxygen as a co-substrate. Additionally, it can reduce heme-bound oxygen to water in the absence or low levels of NO, functioning as NAD(P)H oxidase [25]. It helps *Giardia* withstand the hostile environment and contributes to its persistence and potential drug resistance against metronidazole (MTZ), which is used for giardiasis treatment. Because Flavohemoprotein uses oxygen as a co-factor for converting NO to nitrate, it may also be relevant for increased MTZ tolerance [26, 27].

Giardiasis is a diarrhoeal infection affecting the small intestine of humans and other mammals [28]. Although generally treatable, the effectiveness of the first-line treatment with MTZ is compromised, with success rates ranging between 60% and 80% [29]. The incidence of giardiasis cases refractory to MTZ treatment has been increasing globally [30, 31]. This emerging resistance highlights the need to identify molecular markers for early detection of resistant parasites at diagnosis. Efforts have been made to identify these markers through transcriptomic and proteomic studies; although several potential markers or their combination have been proposed, none have been definitively established. These expression studies were conducted with several MTZ-resistant and MTZ-susceptible cell lines derived in laboratory from sub-assemblage AI strains. Notably, Flavohemoprotein enzyme levels increased during MTZ exposure in 713M3, MTZ-resistant strain [26, 32].

Our interest in one of these potential markers, flavohemoprotein, was sparkled by recent genomic study [33] demonstrating copy numbers variation (CNV) of the *hmpA* gene in *Giardia* clinical isolates belonging to assemblages A and B. It was suggested that its genomic variability may influence the degree of resistance to MTZ. However, this hypothesis has not yet been validated by MTZ treatment outcomes. Similar hypotheses have been tested in other studies. For instance, in *Plasmodium falciparum,* links between CNV and drug resistance have been demonstrated by gene duplications of *mdr1,* which resulted in twofold increases in target gene transcript levels. Increased mRNA expression directly decreased sensitivity to mefloquine, lumefantrine, and dihydroartemisinin [34].

The aim of our study was to evaluate CNV of *hmpA* gene in a cohort of *G. intestinalis* isolates from patients with varying responses to MTZ. We used digital PCR to reveal fine differences in copy numbers among individual *Giardia* isolates, and took advantage from the availability of whole genome sequences for the same collection of isolates.

## Methods

### Samples

A total of 28 DNA samples were extracted from in vitro cultures of *Giardia* parasites (sub-assemblage AII), which were originally isolated from stool samples of human patients between 1989 and 2016. DNA samples were stored at −20 °C until use. Detailed information relevant to this study can be found in Table 1. Specific protocols for the isolation and maintenance of *Giardia* isolates, DNA extraction and genotyping and further characteristics of the samples were described in Lecova et al. [35]. Samples from patients who exhibited treatment failure were classified as clinically MTZ-resistant (referring to the treatment outcome, not in vitro resistance), while samples from patients who tested negative after MTZ treatment were classified as MTZ-sensitive.

**Table 1:** Characteristics of Giardia isolates and their hosts: multilocus genotyping of Giardia, clinical resistance to metronidazole (MTZ), origin of infection, sex of patient and clinical symptoms of giardiasis

*Giardia* isolate	Clinical resistance to MTZ	Multi locus genotype	Sex of patient	Origin of infection	Clinical symptoms
LK16	No	AII-1	M	Sri Lanka	diarrhoea, flatulence, itchy skin
HJ	Yes	AII-1	M	Czech Republic	asymptomatic
37	No	AII-1	F	Argentina	asymptomatic
51	Yes	AII-1	M	Syria	diarrhoea
2	No	AII-2	M	Vietnam	asymptomatic
44	No	AII-3	M	Iran	intermittent diarrhoea
39	No	AII-4	M	Zimbabwe	asymptomatic
12	No	AII-4	M	Libya	diarrhoea
16	Yes	AII-4	M	Czech Republic	asymptomatic
18	No	AII-4	F	Pakistan	asymptomatic
19	No	AII-4	M	Czech Republic	asymptomatic
40	No	AII-4	M	Ethiopia	diarrhoea, fever
41	No	AII-4	F	Ethiopia	asymptomatic
36	No	AII-8	F	Nigeria	asymptomatic
9	No	AII-9	M	Libya	asymptomatic
13	No	AII-9	M	Syria	asymptomatic
8	No	AII-9	M	Vietnam	asymptomatic
20	No	AII-9	M	Kuwait	diarrhoea
23	Yes	AII-9	M	India, Nepal	diarrhoea
24	Yes	AII-9	M	India, Nepal	diarrhoea
25	No	AII-9	F	Vietnam	asymptomatic
28	No	AII-9	M	Czech Republic	mild temperature
34	No	AII-9	M	Czech Republic	slow development
43	No	AII-9	M	Czech Republic	diarrhoea, abdominal pain
50	No	AII-9	M	Sudan	intermittent diarrhoea
52	Yes	AII-9	M	Czech Republic	eczema
53	No	AII-9	M	Indonesia	asymptomatic
BER1	Yes	AII-9	M	Czech Republic	asymptomatic

The second cohort of samples consisted of the isolates from other *Giardia* sub-assemblages. Because this cohort was small in size, it was not used for comparison of MTZ treatment. Additional file S2 contains the list of these samples (*N* = 17): five samples from sub-assemblage AI, five samples from sub-assemblage BIII, four samples from mixed population of sub-assemblages BIII/IV and three samples from sub-assemblage BIV. The DNAs were isolated from cysts obtained from stool samples of human patients with DNaesy PowerSoil Pro Kit (Qiagen) following the manufacturer’s protocol.

### Digital PCR assay designs and optimization

Primers were designed for the *hmpA* (GL50803_15009) and the reference gene β-giardin (*bg*) (GL50803_004812) for *G. intestinalis* assemblage A using Primer BLAST [36]. The second set of primers was designed for assemblage B: for the *hmpA* (GSB_151570) and for the *bg* (GL50581_2741). The criteria for the assays included achieving linearity with a 5-log dynamic range and an LC480 error of < 0.2, along with an efficiency range of 80–100%. The specificity of the primers was confirmed by obtaining single PCR products of the correct sizes.

All assays, each with a 10 µl reaction volume, were tested with SsoFast EvaGreen Supermix (Bio-Rad) on a LightCycler 480 (Roche). The protocol involved initial activation at 95 °C for 30 s, followed by amplification at 95 °C for 5 s and 57 °C for 20 s (for 40 cycles), with a melting curve temperature ranging from 65–95 °C. The annealing temperature, initially set at 60 °C in silico, was experimentally determined to be optimal at 57 °C, along with an optimal final primer molarity of 400 nM.

To assess the performance of the primers for dPCR, they were tested using a Nanoplate 8.5 K with 96 wells and the QIAcuity One instrument (QIAGEN). All assays, each with a 12 µl reaction volume, were evaluated using the QIAcuity EG PCR Kit (QIAGEN). The protocol for this evaluation included activation at 95 °C for 120 s, followed by amplification at 95 °C for 15 s, 57 °C for 15 s and 72 °C for 15 s (for 40 cycles), concluding with a single cycle at 40 °C for 5 min. The optimal annealing temperature for this dPCR assay, where the PCR-positive population of samples was distinguishable from the PCR-negative population of samples, was determined to be 57 °C, along with an optimal final primer concentration of 400 nM.

The 12 µl dPCR mixture consisted of a pair of forward and reverse primers at the final concentration of 400 nM (KRD), 4 µl of 3 × QIAcuity EG PCR Kit (QIAGEN), 3 µl of DNA that was pre-diluted before dPCR, and DNAse-free water. The appropriate DNA dilution for dPCR was established by comparing Cq values obtained from corresponding qPCR experiments conducted on a LightCycler 480 (Roche). The optimization of the dPCR assay and the quality criteria for CNV detection were adopted from the dPCR MIQE guidelines [37]. Information on validation of primers is included in Additional file S1.

### Digital PCR principle in the context of our experiment

The DNA samples, included in a complete dPCR mixture, were pipetted into 96 wells of a dPCR nanoplate (Nanoplate 8.5 K, QIAGEN). We employed well-optimized, singleplex dPCR assays in parallel.

During the dPCR experiment, each sample (from one well) was distributed into up to 8500 nanocompartments (partitions). The central feature of dPCR is the compartmentalization of the DNA sample. In each isolated partition, PCR was performed. Essentially, according to the Poisson distribution, each partition contained a copy of the gene, or did not contain any gene copies, resulting in a zero PCR outcome with no fluorescent signal.

For the detection of positive PCRs, the fluorescent DNA dye Evagreen was used as a single reporter. No-template controls (NTCs) without a sample were included in each nanoplate for each target. Thresholds for each assay were set manually on the basis of the NTC using a 2D scatter plot. Positive and negative PCR reactions were calculated with software, and copy numbers were estimated for each singleplex assay using absolute quantification with the QIAcuity Software Suite 2.1.7.182 (QIAGEN).

Throughout the experiment, particular attention was given to optimizing the template concentration and ensuring optimal distribution of DNA within the nanoplate during the dPCR. The ideal concentration for the gene of interest is 1.6 copies/partition (*λ*), calculated as the ratio of PCR-positive to PCR-negative compartments. Under such conditions, dPCR measurements are the most precise [38]. However, because both targets of interest were measured using the same DNA concentration, achieving optimal saturation of the nanoplate with both positive targets (positive PCRs) was not feasible. To address this issue, specific quality criteria were applied to the data obtained through dPCR, and an optimal and acceptable range for *λ* was defined as 0.1–2.6, corresponding to approximately 10–93% saturation of the nanoplate for both singleplex targets.

### Isolation of DNA for NGS and generation of DNA libraries

Each *Giardia* isolate was cultured to obtain approximately 10^7^ trophozoites, which were subsequently harvested by centrifugation and washed with cold PBS at pH 7.2. The resulting pellet was subjected to DNA extraction. Samples BER1, 16, 18, 19, 20, 23, 24, 25, 28, 34, 36, 40, 41, 43, 44, 50, 52 and 53 were isolated using a modified CTAB method [39]. Samples HJ, LK16, 2, 8, 9, 12, 13, 20, 24, 37, 39 and 51 were isolated using the PureLink^™^ Genomic DNA Mini Kit (Thermo Fisher Scientific); this genomic DNA was subsequently amplified using the Repli-G Midi Kit (Qiagen) following the manufacturer’s instructions. Approximately 1 μg of purified genomic DNA was sent to a commercial sequencing service for NGS experiments. Illumina libraries were generated utilizing the 150 bp paired-end technique and sequenced on an Illumina HiSeq 4000 platform.

### Raw NGS reads for CNV method verification

Raw reads of *Giardia* samples from sub-assemblage AII, including P407 (SRR21529637, SRR21529638, SRR21529640), P64 (SRR21529626, SRR21529627) and P392 (SRR21529641, SRR21529642, SRR21529643), deposited in the NCBI SRA database, along with their assembled and annotated genomes derived from PacBio reads, were generously provided by Christian Klotz [40]. We compared the results obtained from PacBio sequence assemblies with those obtained from an alternative CNV detection approach based on read depth (RD) analysis using Illumina sequencing data from the same *Giardia* isolates. This approach relies on the hypothesis that the read depth in a genomic region is correlated with the copy number of that region. The RD approach is capable of detecting CNVs, including deletions and duplications, and can predict precise copy numbers, mosaicism, and both small and very large CNVs across all types of genomic regions [41–43].

### Gene CNV estimation using NGS data

Raw Illumina reads from 28 isolates, along with reads from three AII samples (P407, P392, P64) retrieved from the NCBI SRA database, were processed by trimming to remove adapter sequences and filtering for low-quality bases using Trimmomatic v.0.39 [44]. The filtered paired reads were aligned to the *Giardia* WBc6 reference genome, sub-assemblage AI [2] using BWA v. 0.7.17 with default parameters [45]. Duplicate reads were identified using Picard tools v.2.26.11 and marked accordingly [46]. Coverage analysis was conducted using Qualimap v.2.2.1 [47] for the *bg* (GL50803_004812) and the *hmpA* (GL50803_0015009) genes, chromosomes and whole-genome regions. Mean coverages, created with the parameter ‘skip duplicate alignments’, were calculated for each sample. The following additional candidate reference genes were subsequently included in the analysis: NADP-specific glutamate dehydrogenase (GL50803_0021942), triosephosphate isomerase (GL50803_0093938) and nitroreductase Fd-NR2 (GL50803_0022677). Coverage ratios were computed as the mean coverage of the region of interest divided by the mean coverage of the entire genome of each *Giardia* isolate (Additional file S3).

### Chromosomal CNV estimation using NGS data

Methods from Bussoti et al. [48], were adopted to assess aneuploidy of chromosomes from *Giardia* isolates, sub-assemblages AII and AI. For each chromosome, we computed the mean sequencing coverage. To account for differences in chromosome copy numbers in tetraploid organisms, all the values were multiplied by four. The differences among the chromosomes were calculated via the Kruskal–Wallis test. Deviation from tetraploidy was calculated for each chromosome by either one-sample *t*-test or one-sample Wilcoxon signed-rank test (Additional file S4).

### Statistical analysis

All the statistical analyses were conducted with either the DATAtab online statistics calculator [49] or the GenEx Enterprise 6.1.1.550 (MultiD). All statistical tests were calculated at the 0.05 level of significance.

## Results

### Impact of genetic variability of *hmpA* on metronidazole treatment outcomes

Digital PCR was utilized for absolute quantification analysis of each gene of interest, which included the *hmpA* and the CNV reference *bg* gene targeting *Giardia* from assemblage A. The copy number estimation was followed by the CNV analysis, in which the results were calculated as the target gene/CNV reference gene ratio and subsequently reported as the number of *hmpA* copies per haploid genome. For accurate analysis of the CNV, the reference gene must have a known copy number, ideally being a single-copy gene. Digital PCR CNV analysis yields the ratio of concentrations (copies/µL) of the target and reference genes, along with their associated CI (95%), for each *Giardia* isolate (Additional file S2).

The CNVs of the *hmpA* gene in *Giardia* isolates, collected from patients who underwent MTZ treatment, were analysed. In total, 28 DNA samples of *G. intestinalis,* sub-assemblage AII, were examined, representing clinically MTZ-resistant (7 samples) or MTZ-susceptible (21 samples) cases of giardiasis (Table 1). Our aim was to find out whether differences in the *hmpA* gene copy numbers of the parasite correlated with the treatment resistance.

**Fig. 1:**
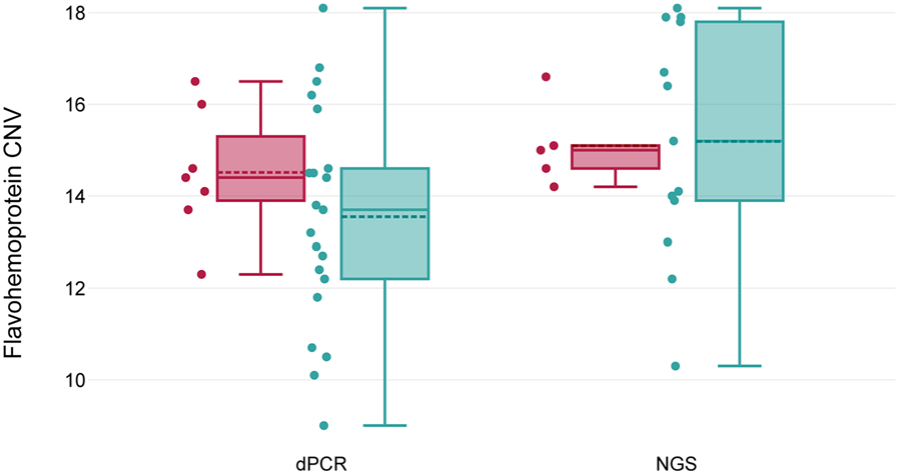
Flavohemoprotein copy number variations (CNVs) were estimated using different methods. The green box plots represent the copy numbers of samples sensitive to metronidazole treatment, and the red box plots represent those with the clinical resistance to metronidazole treatment. Digital PCR CNV analysis (*N* = 28) and NGS CNV analysis (*N* = 18). CNVs were recalculated per tetraploid genome

The mean *hmpA* CNV for clinically MTZ-resistant samples was estimated to be 3.6 ± 0.3 copies per haploid genome, and 3.4 ± 0.6 copies per haploid genome for MTZ-sensitive samples. These copy numbers were recalculated for tetraploid genome of *Giardia* in Fig. 1. The dPCR analysis revealed no difference between MTZ-sensitive and clinically MTZ-resistant samples, which was confirmed with a *t*-test for independent, normally distributed samples (*t*_(26)_ = −0.98, *P* = 0.3). The copy numbers of *hmpA* from the entire cohort of patients, measured by dPCR, ranged from 9 copies to 18 copies when recalculated per tetraploid genome.﻿﻿﻿

### Validation of dPCR results using parallel NGS methodology

Prior to the comparative analysis itself, we tested the reliability of the selected single-copy reference gene (*bg*) for the CNV analysis. We used three previously published genomes of *G. intestinalis* belonging to sub-assemblage AII (P407, P392 and P64), which were acquired via both PacBio and Illumina platforms. PacBio results confirmed that *bg* was present only once in each tested genome, consistently with findings from Illumina. In contrast, the *hmpA* gene was located in multiple sites, aligning with the Illumina results (Additional file 3). These results supported the use of *bg* as a suitable CNV reference gene for dPCR.

Subsequently, the same samples used for dPCR were utilized for NGS analysis. All NGS data for CNV assessment were obtained via Illumina-based whole-genome sequencing. For NGS CNV estimation, only *hmpA* gene is needed. It is important to note that the NGS CNV analysis differs substantially from the dPCR CNV analysis; NGS does not require a CNV reference gene, as the target *hmpA* gene is mapped directly against the reference genome AI (WBc6).

Despite these methodological differences, the *bg* gene selected for dPCR was also analysed using NGS to verify the reliability of our approach. Interestingly, our findings revealed some discrepancies when comparing dPCR with NGS results. In approximately one-third of the AII samples analysed by NGS, the coverage depth in the *bg* region was found to be 50% lower than the expected coverage depth of the *bg* region of the AI reference genome. In the same samples, the mean copy numbers of *hmpA* also differed when compared with dPCR results (Table 2).

**Table 2:** Copy number variations (CNVs) of flavohemoprotein (subassemblage AII) estimated with different methods and copy numbers (CNs) of candidate CNV reference genes for digital PCR (dPCR)

*G. intestinalis* isolates/genes	dPCR CN *hmpA*	WGA for NGS only	NGS CN *hmpA* (chr. 5)	NGS CN *tpi* (chr. 5)	NGS CN *gdh* (chr. 4)	NGS CN *bg* (chr. 4)	NGS CN *fd-nr2* (chr. 1)
HJ	3.5	Yes	0.9	1.1	0.6	0.5	1.0
LK16	3.4	Yes	1.0	0.8	0.4	0.6	1.0
2	4.1	Yes	1.3	0.8	0.5	0.5	1.1
8	2.9	Yes	1.0	0.9	0.5	0.5	1.0
9	2.3	Yes	1.0	0.9	0.5	0.5	1.0
12	4.5	Yes	1.0	0.9	0.5	0.4	1.0
13	2.5	Yes	0.8	0.9	0.5	0.5	1.0
20	N/A	Yes	1.1	0.8	0.5	0.5	1.3
24	N/A	Yes	1.2	0.8	0.5	0.5	1.1
37	3.3	Yes	1.3	1.0	0.6	0.5	1.0
39	2.7	Yes	0.8	1.0	0.6	0.5	1.1
51	4.1	Yes	1.5	0.9	0.6	0.6	1.0
MTZ resistant *N* = 3 avg CN ± std		WGA	1.2 ± 0.2	0.9 ± 0.1	0.6 ± 0.05	0.5 ± 0.05	1.0 ± 0.05
MTZ sensitive *N* = 9 avg CN ± std		WGA	1.1 ± 0.3	0.9 ± 0.2	0.5 ± 0.1	0.5 ± 0.1	1.1 ± 0.1
BER1	3.1	No	3.6	1.0	1.0	1.0	1.0
16	3.4	No	3.6	1.0	1.0	1.1	1.0
18	3.4	No	3.1	0.9	1.0	1.0	1.0
19	3.1	No	3.5	1.0	1.0	1.0	1.0
20	3.6	No	3.8	0.9	1.0	0.9	1.0
23	3.6	No	3.8	0.9	0.9	1.0	1.0
24	3.6	No	3.8	0.9	1.0	0.9	1.0
25	3.6	No	2.6	1.0	1.0	1.0	1.0
28	3.1	No	4.2	1.0	1.0	1.0	1.2
34	3.6	No	4.5	1.0	1.1	1.1	1.1
36	4.2	No	4.4	1.0	1.0	1.0	1.1
40	2.6	No	3.2	1.0	1.2	1.2	1.2
41	3.2	No	3.5	1.0	1.0	1.0	1.1
43	4.0	No	4.5	1.0	1.0	0.9	1.0
44	3.6	No	4.1	1.0	1.0	1.0	1.0
50	3.2	No	3.5	1.0	1.0	1.0	1.0
52	4.0	No	4.1	1.0	1.0	1.0	1.1
53	4.1	No	4.5	1.0	1.0	1.0	1.0
MTZ resistant *N* = 5 avg CN ± std	3.5 ± 0.3	w/o WGA	3.8 ± 0.2	1.0 ± 0.05	1.0 ± 0	1.0 ± 0.1	1.0 ± 0.04
MTZ sensitive *N* = 13 avg CN ± std	3.5 ± 0.4	w/o WGA	3.8 ± 0.6	1.0 ± 0.04	1.0. ± 0.1	1.0 ± 0.1	1.0 ± 0.1

The CNs values were averaged ± standard deviation. CNs were recalculated per haploid genome. *chr.* chromosome, *dPCR* digital PCR, *MTZ* metronidazole, *NGS* next-generation sequencing, *WGA* whole genome amplification

### The whole genome amplification in NGS: a troublesome variable

We discovered that discrepancies in results could be linked to the whole genome amplification (WGA) of DNA samples prior to NGS analysis. To further investigate this, we analysed three additional single-copy genes, using NGS data: the glutamate dehydrogenase gene (*gdh*), located on chromosome 4, the same location as *bg*; the triosephosphate isomerase gene (*tpi*), located on chromosome 5, the same location as *hmpA*; and the nitroreductase Fd-NR2 gene (*fd-nr2*), located on chromosome 1. The coverage depths of all analysed genes are detailed in Table 2. The results for both the *bg* and *gdh* genes, expected to be located on the same chromosome 4, showed lower coverage depth ratios within the same *Giardia* isolates. Additionally, lower *bg* coverage depth ratios were consistently associated with lower *hmpA* coverage depth ratios, despite these genes being located on separate chromosomes. In contrast, both the *tpi* and the *fd-nr2* genes (located on chromosome 5 and 1, respectively) were not affected by WGA.

The inconsistency and non-uniformity of the whole genome amplification were further explored through an additional experiment comparing samples 20 and 24, which underwent different conditions: either WGA amplification or no pre-amplification prior to NGS analysis (Additional file S3). The results from samples 20 and 24 were entirely consistent with previous observations, showing differences comparable to those observed in other samples reported in Table 2. On the basis of these findings, all samples that underwent WGA were excluded from the validation NGS CNV analyses.

### Validation of dPCR data with NGS data

After the exclusion of NGS WGA samples and their corresponding dPCR samples, the remaining *hmpA* CNV results obtained via dPCR and NGS showed close agreement. The mean *hmpA* CNV for all dPCR analyses was 3.5 ± 0.4 copies per haploid genome (*N* = 18), compared with 3.8 ± 0.5 copies per haploid genome (*N* = 18) for all NGS analyses (Table 2).

The mean *hmpA* CNV for clinically MTZ-resistant samples was estimated to be 3.8 ± 0.2 copies per haploid genome as well as for MTZ-sensitive samples; this value was 3.8 ± 0.6 copies per haploid genome (Fig. 1). The *hmpA* CNVs estimated with NGS demonstrated no significant difference in gene copy numbers between clinically MTZ-resistant and MTZ-sensitive *Giardia* isolates (Table 2). NGS results were statistically evaluated by a two-tailed *t*-test for independent samples, which were distributed normally with not equal variances (*t*_(15.92)_ = 0.1, *P* = 0.9).

When recalculated, the range of *hmpA* copy numbers among different *Giardia* isolates varied from 10 to 18 copies per tetraploid genome according to NGS CNV analysis. The dPCR and NGS results were in agreement, thus dPCR was considered validated by NGS method for our purposes.

### Variation in the number of chromosomes

**Fig. 2:**
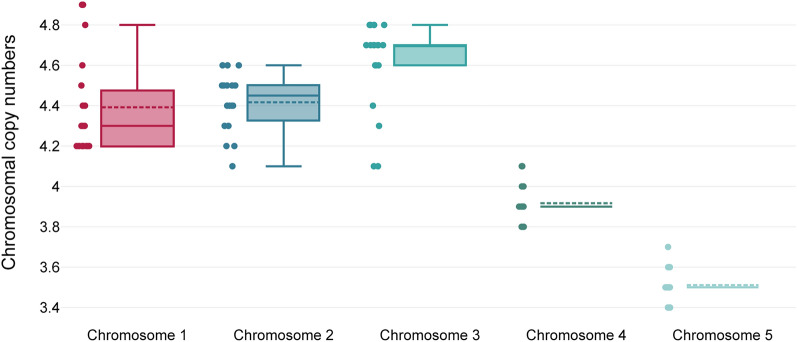
Variations in chromosomal copy numbers in the tetraploid genome of *Giardia*, sub-assemblage AII. *N* = 18

We considered the possibility that the CNV of certain genes might be influenced by the variability of individual chromosomal numbers. To explore this in the sub-assemblage AII, we utilized NGS data to analyse chromosomal copies. This analysis involved assessing RD coverage values, which corresponded to chromosomal copy numbers. The results from the respective chromosomes are displayed in Fig. 2. Our findings revealed the presence of mosaic aneuploidy, with varying numbers of chromosomes present in *Giardia* isolates from sub-assemblage AII. The differences in the number of chromosomes among five different chromosomes were confirmed by the Friedman test: *χ*^2^ = 59.4, *df* = 4, *P* < 0.001.

To assess the potential aneuploidy of chromosomes, one-sample tests were performed. One-sample *t*-tests for chromosomes 1, 2 and 3 showed significant deviation of chromosomal numbers from tetraploidy, with *t*-values of 6.8, 12.1 and 11.3, respectively, all with *P*-values less than 0.001. The mean differences were 0.4 for chromosome 1, 0.4 for chromosome 2 and 0.6 for chromosome 3, each with tight confidence intervals indicating precise estimates. For chromosomes 4 and 5, one-sample Wilcoxon Signed-rank tests were performed, yielding *z*-values of −3.5 and −3.8, respectively, both with *P*-values below 0.001, confirming significant differences in these chromosomes as well. These tests confirmed that all chromosomes differed significantly from tetraploidy.

Chromosomes 1, 2 and 3 exhibited tendencies for chromosomal gain, each fluctuating between a minimum of 4 and a maximum of 5 chromosomes per a cell: chromosome 1 (mean: 4.4, variance: 0.06), chromosome 2 (mean: 4.4, variance: 0.02) and chromosome 3 (mean: 4.6, variance: 0.05) In contrast, chromosome 5, while showing a predisposition for losses, demonstrated considerable stability, maintaining between 3 and 4 copies per cell with a low variance (mean: 3.5, variance: 0.01). This underscores its relative stability despite the observed range. The copy numbers for chromosome 4 were notably stable, consistently maintaining four copies in most isolates with a low variance (mean: 3.9, variance: 0.01). The low variance for both chromosomes 4 and 5 indicates minimal fluctuation in the number of copies around their mean (Additional file S4).

### MTZ resistance in *Giardia* isolates AII: a comprehensive multivariate analysis

In an effort to understand why some *Giardia* isolates resist MTZ treatment, we integrated results from our dPCR experiments (*hmpA* CNV) and NGS experiments (chromosomal copy numbers) with data extracted from patient records. These records encompassed variables such as geographic origin of infection, symptoms, sex and the parasite’s response to MTZ treatment (Table 1).

**Fig. 3:**
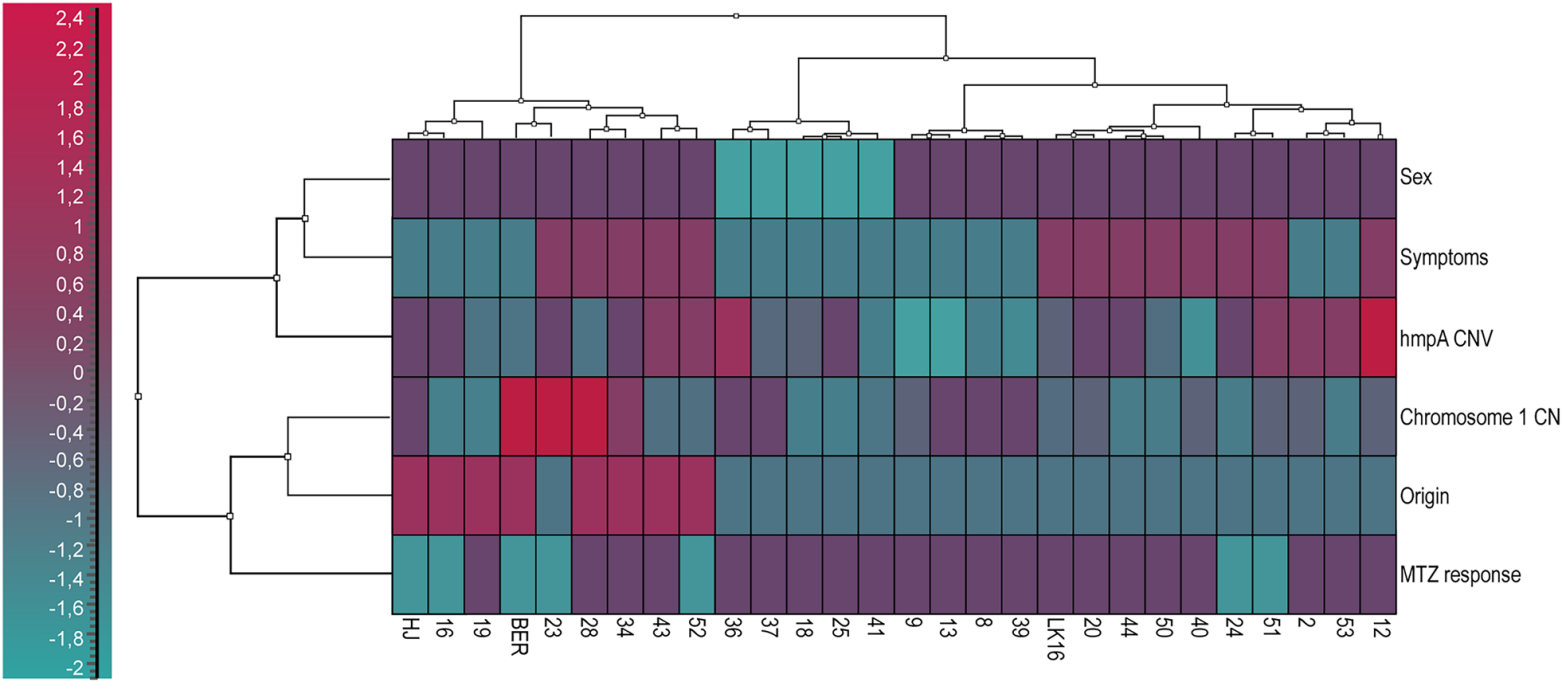
The cluster analysis: a heat map is a way of visualizing hierarchical agglomerate clustering. Ward’s algorithm clustering method and the Euclidean distance measure were applied

To visualise the relationships between selected variables and MTZ resistance, the cluster analysis was employed. The data were autoscaled to ensure equal weighting of all variables in the analysis. For the cluster analysis in Fig. 3, Ward’s algorithm clustering method with the Euclidean distance measure was used. It did not reveal distinct groupings between clinically MTZ-resistant and MTZ-sensitive *Giardia* isolates, despite incorporating multiple variables. It means that although groups based on the analysed variables were formed, they overlap and there is no clear grouping that consistently indicates resistance to MTZ. This lack of distinct groupings suggests that resistance may not be strongly linked to the genetic variations captured by the CNV of *hmpA*, nor is it clearly associated with demographic or clinical characteristics. Furthermore, while our analysis sheds light on certain aspects of MTZ resistance, it also indicates that better understanding of the MTZ resistance likely requires additional factors beyond those included in our study.

### Flavohemoprotein CNVs in various *Giardia* sub-assemblages

**Fig. 4:**
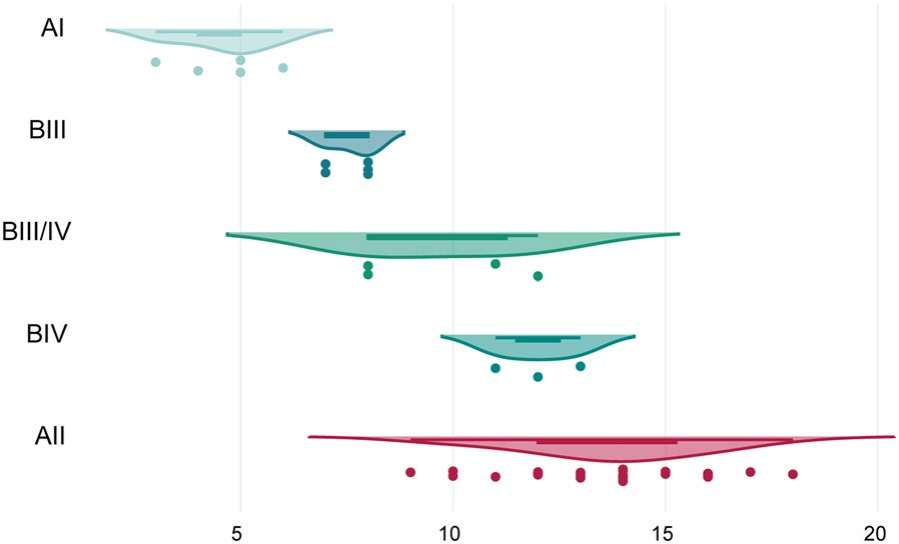
Distribution of the mean flavohemoprotein copy number variations (CNVs) among different *Giardia* sub-assemblages: AI (CN = 4.8 ± 1.2, *N* = 5), AII (CN = 13.6 ± 2.0, *N* = 28), BIII (CN = 8.0 ± 0.8, *N* = 5), BIV (CN = 12.0 ± 0.8, *N* = 3) and BIII/IV (CN = 10.0 ± 2.14, *N* = 4). CNVs were recalculated per tetraploid genome (plotted on x-axis)

Finally, we expanded our analysis of *hmpA* CNVs by including additional *Giardia* sub-assemblages AI, BIII, BIII/IV, and BIV, using dPCR. Due to the limited sample sizes for these sub-assemblages, a comparative analysis between MTZ-sensitive and clinically MTZ-resistant isolates was not performed. Instead, the CNVs were measured exclusively to observe potential variations across sub-assemblages. As it is displayed in Fig. 4, the differences in copy numbers of *hmpA* among sub-assemblages were significant according to Kruskal–Wallis *χ*^2^ test; *χ*^2^ = 28.3, *df* = 4, *P* < 0.001.

DNA isolates representing sub-assemblage AI displayed the lowest mean copy numbers of the *hmpA* gene (1.2 ± 0.3 copies per haploid genome), significantly lower than those recorded in sub-assemblages AII (3.4 ± 0.5 copies per haploid genome) and BIII (2.0 ± 0.2 copies per haploid genome), as confirmed by Dunn–Bonferroni post-test (AII–AI: *t* = 27.5, adj. *P* < 0.001; AII–BIII: *t* = 21.5, adj. *P* = 0.007). The highest mean copy numbers were observed in sub-assemblage AII, closely followed by BIV (3.0 ± 0.2 copies per haploid genome) and BIII/IV (2.5 ± 0.6 copies per haploid genome). Sub-assemblage BIII exhibited intermediate values with lower variability in copy numbers. Despite the statistically significant differences found between sub-assemblages, caution should be exercised when interpreting these results due to the small sample sizes of the groups involved.

## Discussion

The aim of this study was to enhance our understanding of the multi-copy *hmpA* gene. Multi-copy genes are typically involved in critical cellular processes, such as survival and adaptation to various environments, as has been well documented in extensive research on *hmp* in bacteria and in other single-celled organisms [24].

Here, we focused specifically on *hmpA* CNV in *G. intestinalis*. To estimate CNVs, we used two methods: dPCR CNV analysis [50, 51] and a RD-based NGS CNV approach, which has been widely used in previous studies [42, 48, 52]. After appropriate adjustments, both methods produced consistent results and validated each other. These adjustments included revising the WGA incorporation for NGS analysis. WGA is generally used for the entire genome amplification to ensure sufficient material is available for NGS [53]. Despite information suggesting that the whole genome amplification with Repli-G kit is suitable for CNV detection in single cells [54], our findings suggested otherwise. Due to uneven amplification of our genes of interest, we had to exclude ten isolates from validation experiments.

With the remaining subset of NGS samples, that were not treated with WGA, we explored the presence of chromosomal aneuploidy, which means the variation in chromosome numbers from the standard set. Our findings confirmed the presence of mosaic aneuploidy, which might complicate CNV analysis. However, chromosomes 4 and 5, where our genes of interest were located, exhibited very low variance in their copy numbers across different isolates. This stability minimized the potential impact of chromosomal aneuploidy on our CNV measurements, allowing us to accurately assess the role of *hmpA* in relation to MTZ resistance. Aneuploidy is a concept that has been often overlooked in *Giardia*, despite previous studies highlighting the genetic instability of chromosome 5 (sub-assemblage AI) and the presence of cell lines with unstable aneuploidy as well as those with long-term stable aneuploid karyotypes [4, 5]. Among unicellular parasites, aneuploidy has been well-studied in *Leishmania donovani*, a diploid organism with a single nucleus. Karyotypic changes in different *Leishmania* strains have shown reproducible variations in the same subset of chromosomes, connected to short-term adaptations to environmental changes during the parasite’s life cycle in in vitro culture [48]. It is likely that a combination of chromosome and gene amplifications (or loss) generates phenotypic diversity enhancing parasite fitness [55, 56]. These assumptions may also be valid for *Giardia*. The effects of aneuploidy in unicellular organisms can vary depending on the specific organism and the chromosomes involved.

Using the dPCR method, we extended the investigation of *hmpA* CNVs to *Giardia* sub-assemblages beyond sub-assemblage AII. By comparing sub-assemblages AI, AII, BIII, mixed population BIII/IV and BIV, we revealed significant differences in *hmpA* CNVs across these sub-assemblages. Isolates from sub-assemblage AI exhibited the lowest *hmpA* copy numbers, suggesting that *hmpA* exists in a single-copy form, in contrast to the other tested *Giardia* isolates from different sub-assemblages. This finding is not surprising given that the overall genetic diversity of the sub-assemblage AI is low if compared with other sub-assemblages. This has been demonstrated by its allelic sequence heterozygosity (ASH), which is as low as 0.03% in sub-assemblage AI reference genome [1]. Reported ASHs of other assemblages and sub-assemblages were notably higher. The ASH in the assemblage B isolate GS reference genome is about 20-fold higher than in assemblage A reference genome WBc6 [1], although an exception, such as B isolate P424 with extremely low ASH = 0.002%, has been discovered [40]. A higher ASH for sub-assemblage AII ranges from 0.25% to 0.35% [9]. These observations correlate with our findings that all other tested isolates from other sub-assemblages and assemblages contained multicopy variants of *hmpA*. Interestingly, we showed that isolates from sub-assemblage AII consistently showed the highest mean *hmpA* copy numbers (14 copies per tetraploid genome) compared with sub-assemblage AI and assemblage B. These observations underscore the genetic diversity within *Giardia* and imply that different sub-assemblages might exhibit varying degrees of adaptability strategies.

On the basis of observed genetic diversity, we decided to reduce unnecessary variability in our main experiments by focussing only on assemblage A, namely sub-assemblage AII [16], by excluding other known sub-assemblages (AI, BIII, BIV) [15]. The selection of AII was also supported with its unique genetic characteristics (notably higher ASH) as well as previous observations of significant genetic variability not only between described assemblages or sub-assemblages, but also within individual sub-assemblages [57]. By focussing only on AII sub-assemblage, together with usage of the large group of clinically described MTZ-sensitive and MTZ-resistant giardiasis cases characterized by NGS, we aimed to create more controlled experimental setting. The limited confounding variability could help to unravel the precise relationship between genetic variations and drug resistance in *Giardia* and to find out whether genomic variations in copy numbers of the multi-copy *hmpA* gene could be linked to *Giardia*’s adaptability to MTZ toxicity. With both methods, dPCR and NGS, we confirmed that CNV of *hmpA* does not play a significant role in protecting *Giardia* against MTZ treatment despite the previous suggestions. We can say that the differences in *hmpA* copy numbers between MTZ-sensitive and clinically MTZ-resistant isolates within sub-assemblage AII are less pronounced than those observed between different sub-assemblages.

Regrettably, no reliable markers for *Giardia*’s resistance to MTZ have been identified yet [26, 32], and here, we added further evidence that neither CNV of *hmpA* can serve as a marker of MTZ resistance. Resistance in parasites such as *Giardia* and other microorganisms is often multifactorial and complex, making it unlikely to be associated with a single specific marker. The preferred method for detecting MTZ resistance would be a multivariate approach capable of distinguishing differences in samples on the basis of a specific set of markers, creating a diagnostic profile in which each marker plays a partial role. We attempted to introduce this approach; however, we lacked sufficient number of variables to reliably differentiate MTZ-refractory cases from MTZ-sensitive cases.

## Conclusions

*G. intestinalis* displays substantial genetic and genomic diversity within its assemblages and sub-assemblages. This study focused on *Giardia* AII isolates and aimed to investigate genetic and genomic variations associated with clinical MTZ resistance. Despite efforts to link CNV of the *hmpA* gene to MTZ resistance, our comprehensive analysis did not reveal an association. Our study has elucidated several important aspects of the flavohemoprotein gene in *G. intestinalis*:

- The presence of variable *hmpA* copy numbers does not influence the clinical response of *Giardia* to MTZ treatment, as there was no significant difference in these numbers between MTZ-resistant and MTZ-sensitive isolates within *Giardia* sub-assemblage AII.
- The significant variations in the flavohemoprotein copy numbers among different sub-assemblages were observed, with flavohemoprotein consistently present as a multicopy gene, except in sub-assemblage AI where it appears as a single copy gene.

Other information revealed:

(iii) The mosaic aneuploidy in *Giardia*, particularly within sub-assemblage AII, has been described. This aspect necessitates careful consideration in the design of CNV studies, especially when employing dPCR, as it poses challenges in selecting appropriate CNV reference genes.
(iv) By using both dPCR and NGS methodologies, we were able to reciprocally validate our findings and identify potential issues fast. The validation of the results was a key step for ensuring the reliability of our CNV analysis.
(v) The whole genome amplification proved to be unsuitable for CNV analysis of our genes of interest, as the amplification across different regions of the genome was not even.
(vi) This study underscores the complexity of MTZ resistance in *Giardia*, where reliable markers have yet to be identified.

### Supplementary Information

Additional file 1: S1. Primer design and validation of assays: Information on primers, PCR products, qPCR protocol, dPCR protocol, assay efficiency, dPCR quality and dPCR repeatability.

Additional file 2: S2. Digital PCR data and CNV estimation of genes of interest and dPCR analysis of digested and undigested DNA.

Additional file 3: S3. NGS coverage data and CNV estimation of genes of interest. Comparison of WGA and nonWGA data. Analysis of test samples for evaluation via the CNV NGS method.

Additional file 4: S4. NGS coverage data for the estimation of chromosomal copy numbers and statistical tests for determination of ploidy, descriptive statistics and aneuploidy tests.

## Data Availability

Minimal datasets that are necessary to interpret results reported in the manuscript are included in the supplementary information part of the manuscript. Relevant raw NGS data will be freely available to any scientist wishing to use them for non-commercial purposes without breaching participant confidentiality.

## Abbreviations

ANOVA: Analysis of variance
*bg*: β-Giardin gene
CI: Confidence interval
CN: Copy number
CNV: Copy number variation
CTAB: Cetyltrimethylammonium bromide
dPCR: Digital polymerase chain reaction
EG: EvaGreen dye
*fd-nr2*: Fd-NR2 gene
G1 and G2: Cell cycle checkpoints
*gdh*: Glutamate dehydrogenase gene
*hmpA*: Flavohemoprotein gene
λ: Number of gene copies per partition in dPCR
LC480: qPCR instrument LightCycler 480
Mb: Megabyte
MIQE: Minimum information for publication of quantitative real-time PCR experiments
MTZ: Metronidazole
NCBI: The National Center for Biotechnology Information
NGS: Next generation sequencing
NO: Nitric oxide
NTC: No template control
PBS: Phosphate-buffered saline buffer solution
PCA: Principal component analysis
qPCR: Quantitative polymerase chain reaction
RD: Read depth method
SRA: Sequence read archive
*tpi*: Triosephosphate isomerase gene
VSP: Variable surface protein
WGA: Whole genome amplification
